# Systemic signals in aged males exert potent rejuvenating effects on the ovarian follicle reserve in mammalian females

**DOI:** 10.18632/aging.100255

**Published:** 2010-12-31

**Authors:** Yuichi Niikura, Teruko Niikura, Ning Wang, Chonthicha Satirapod, Jonathan L. Tilly

**Affiliations:** Vincent Center for Reproductive Biology, Vincent Department of Obstetrics and Gynecology, Massachusetts General Hospital/Harvard Medical School, Boston, MA 02114, USA

**Keywords:** aging, germ cell, oogenesis, oocyte, ovary, stem cell, parabiosis

## Abstract

Through the use of parabiosis in mice, aging-related deterioration of skeletal muscle and liver has been linked to a loss of systemic factors that support adult stem or progenitor cell activity. Since aging-related ovarian failure has recently been attributed, at least in part, to a loss of de-novo oocyte-containing follicle formation associated with declining oogonial stem cell activity, herein we tested in mice if aging-related changes in systemic factors influence the size of the ovarian follicle reserve. Ovaries of young (2-month-old) females parabiotically joined with young females for 5 weeks possess comparable numbers of healthy and degenerative (atretic) oocyte-containing follicles in their ovaries as those detected in non-parabiotic young females. Joining of young females with young males significantly increases follicle atresia without a net change healthy follicle numbers. Surprisingly, young females joined with aged (24-month-old) males exhibit a significant increase in the number of primordial follicles comprising the ovarian reserve, and this occurs without changes in follicle growth activation or atresia. Blood of aged males also induces ovarian expression of the germ cell-specific meiosis gene, Stimulated by retinoic acid gene 8 (Stra8), in ovaries of female parabionts, further supporting the conclusion that the observed changes in the follicle reserve of females joined with aged males reflect increased oocyte formation. Thus, factors in male blood exert dramatic effects on ovarian follicle dynamics, and aging males possess a beneficial systemic factor that significantly expands the ovarian follicle reserve in females through enhanced oogenesis.

## INTRODUCTION

The contribution of declining stem cell function to organismal aging has gained widespread interest in recent years [1]. For example, genetic studies in mice have demonstrated that the senescence gene locus, *Ink4a*, is a negative regulator of adult stem cell regenerative capacity in the aging heart, brain and haematopoietic system [2-4]. While these findings support a role for stem cell-intrinsic changes during aging, evidence derived from parabiosis - or the joining of two animals to produce a shared circulatory system - has revealed that extrinsic systemic signals also contribute to stem cell fate determination with advancing age [5]. For example, circulating factors present in young mice effectively restore Notch signaling and the regenerative capacity of skeletal muscle satellite cells in aged parabionts [5]. Similar beneficial effects were noted in the liver, leading to the conclusion that stem or progenitor cells in diverse tissues of aged animals can be rejuvenated by exposure to a young systemic environment [5].

Herein we sought to further test and extend this concept using the female gonads as a model system. In mammals, age-related failure of the ovaries, which in humans leads to menopause [6], is driven by progressive depletion of oocyte-containing follicles throughout postnatal life [7]. Only a small number of the quiescent primordial follicles that comprise the ovarian reserve successfully complete growth through primary and preantral stages of development to yield maturing antral follicles for ovulatory selection. The vast majority of follicles will at some point succumb to a degenerative process referred to as atresia. It was assumed for decades that the primordial follicle reserve is non-renewing due to disappearance of oocyte-producing stem cells from the ovaries before birth [8]. However, recent studies [9], including isolation of germline stem cells from mouse ovaries that can generate oocytes in vitro [10] and developmentally-competent eggs in vivo [11], have essentially invalidated this longstanding dogma (reviewed in [12, 13]). Hence, efforts to delineate factors that regulate the activity of oocyte-producing (oogonial) stem cells, and how these events dictate the size of the ovarian follicle reserve during adulthood, are critical for progress in this new field.

## RESULTS

### Heterochronic parabiosis uncovers an ovarian rejuvenating activity in males

**Figure 1.**
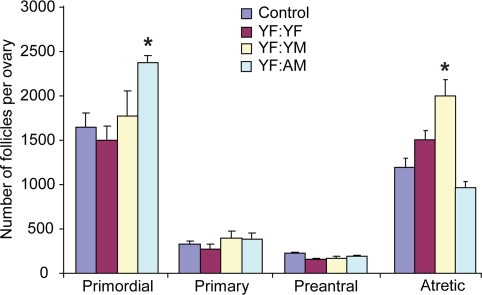
Blood of aged males increases the ovarian follicle reserve in adult females Non-atretic and atretic immature follicle numbers in ovaries of control (non-parabiotic) YF mice or YF mice 5 weeks after parabiosis with YF, YM or AM mice. Data are the mean ± SEM (n = 3-4 mice per group; *, *P* < 0.05).

In initial experiments, 2-month-old female (young female, YF) mice were parabiotically joined with YF mice, 2-month-old male (young male, YM) mice or 24-month-old male (aged male, AM) mice. Since we recently reported that ovarian follicle dynamics in YF mice are unaffected after parabiotic joining with aged females [14], this pair group was not included again for analysis here. Ovaries of YF mice joined with YF mice for 5 weeks possessed comparable numbers of morphologically healthy (non-atretic) and degenerative (atretic) follicles as those detected in non-parabiotic YF controls (Figure 1). Joining of YF mice with YM mice for 5 weeks significantly (*P* = 0.007) increased the incidence of follicle atresia, without a corresponding decline in the number of healthy primordial or total immature follicles. Surprisingly, ovaries of YF mice joined with AM mice for 5 weeks exhibited a significant (*P* = 0.013) increase in primordial follicle numbers (Figure 1). Under the dogma that this follicle reserve in mammals is not subject to renewal [8], there are only two explanations for this outcome. Systemic factors present in blood of AM mice either reduce the rate of primordial follicle growth activation to the primary stage of development (which would be manifest by reduced primary follicle numbers) or decrease follicle loss through atresia. However, the increase in primordial follicle numbers in YF mice resulting from exposure to AM blood was observed in the absence of any change in the number of healthy primary (or preantral) follicles and with no change in the incidence of atresia (Figure 1). These findings therefore leave only one other possible explanation for the increase in primordial follicle numbers that occurs in ovaries exposed to the blood of aged males, that being enhanced oogenesis and folliculogenesis.

**Figure 2.**
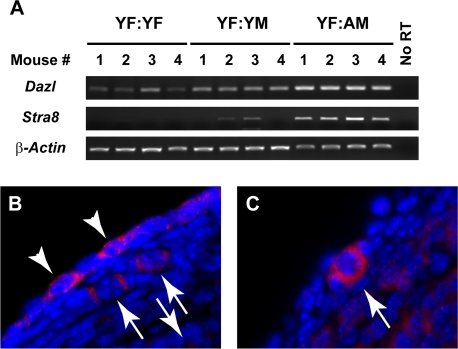
Ovarian *Stra8* expression is increased by exposure to blood of aged males (**A**) Analysis of *Dazl*, *Stra8* and *β-actin* mRNA levels in ovaries of YF mice 5 weeks after parabiosis with YF, YM or AM mice (n = 4 mice per group, with results from each mouse shown; no RT, RNA without reverse transcriptase, used as a negative control). (**B, C**) Examples of STRA8-positive cells in ovaries of YF mice 5 weeks after parabiotic joining with AM mice, as detected by immunofluorescence (red immunopositive signal against blue DAPI counterstain).

### Blood of aged males induces ovarian *Stra8* expression in females

In light of the findings above, we further tested the possibility that male blood contains a factor that stimulates formation of new primordial follicles. Our approach was based on recent studies showing that two different histone deacetylase inhibitors, trichostatin-A and suberoylanilide hydroxamic acid, increase primordial follicle numbers in adult female mice through denovo oogenesis [15, 16]. This response involves induction of *Stimulated by retinoic acid gene 8* (*Stra8*) [16], a germ cell-specific gene that both marks and is required for meiotic commitment associated with oocyte formation [17-21]. Analysis of *Stra8* in ovaries of YF mice from the three parabiotic pair groups indicated that expression of the gene was barely, if at all, detectable after joining with YF mice; however, increased expressed of *Stra8* was detected in ovaries of YF mice paired with either YM or AM mice, and the magnitude of this response was age-dependent (Figure 2A). Analysis of *Dazl*(*Deleted in azoospermia-like*), a second germ cell-specific gene involved in meiotic initiation that remains detectable in oocytes once formed [22, 23], showed a similar pattern of elevated expression in YF ovaries exposed to male blood (Figure 2A). Parallel immunohistochemical assessment of ovaries of YF mice joined with AM mice revealed the appearance of STRA8-positive cells that were either not enclosed by somatic granulosa cells as follicles (Figure 2B: white arrowheads) or appeared as oocytes contained within primordial follicles (Figure 2B, C: white arrows). As recently reported [14, 16], STRA8-immunopositive cells were rarely detected in ovaries of YF controls (data not shown).

## DISCUSSION

This study has uncovered an unexpected rejuvenating effect of aged male blood on ovarian follicular dynamics in adult female mice. Both young and aged males appear to possess this activity in their systemic circulation. However, the beneficial effects of this activity on increased primordial follicle formation in YF mice joined with YM mice is offset by increased follicle atresia, leading to no net change in the ovarian reserve. In contrast, the atresia-inducing activity of male blood is lost with age, leaving only a stimulatory effect of AM blood on increasing the size of the follicle reserve. There are several ramifications of these findings. First, aging of the ovaries, which is defined by depletion of the follicle reserve, is a process that begins at birth [8, 9]. Past estimates of oocyte numbers in humans indicate that well over one-half of the follicles present in neonatal ovaries are lost by puberty, and the remaining undergo a steady if not accelerated decline until exhaustion at menopause [24-27]. Identification of factors that can replenish the ovarian reserve, even during young adulthood, opens the prospects for development of new anti-aging strategies. Second, alterations in availability of systemic factors with age do not uniformly equate to a disruption of adult stem cell function or tissue homeostasis, as has recently been concluded from studies of skeletal muscle and liver in parabiotic animals [5]. Our experiments have also shown that sex-mismatched parabiosis is a valuable model for testing how sex-specific changes in systemic factors with age can influence stem cell and organ function. Finally, our finding of a *Stra8*-inducing activity in the circulation of males is important not only for highlighting the need to expand studies of meiosis from gonadal microenvironments to include systemic analyses, but also for identifying potentially novel regulators of germ cell fate determination in mammals.

## METHODS

### Animals.

Wild-type C57BL/6 mice were obtained from the National Institute on Aging (Bethesda, MD) and Jackson Laboratories (Bar Harbor, ME). All procedures reported herein were approved by the institutional animal care and use committee of Massachusetts General Hospital

Parabiosis. Mice were surgically joined for parabiosis [14], and ovaries were removed from YF mice 5 weeks later. From each mouse, one ovary was processed for follicle counts whereas the other ovary was bisected for RT-PCR analysis (one half) and STRA8 immunodetection (other half).

Follicle counts. The number of non-atretic and atretic immature (primordial, primary and preantral) follicles per ovary was determined by histomorphometry [28].

### Gene expression analysis.

*Stra8* and *Dazl* mRNA levels were assessed by RT-PCR using β*-actin* mRNA as a control, as detailed previously [14, 16].

### STRA8 immunodetection.

Ovarian sections were analyzed by immunofluorescence for the presence of STRA8, as detailed [14].

### Data presentation and analysis.

Experiments were independently replicated 3-4 times, using different pairs of mice for each experimental replicate. Data from the replicate experiments were pooled and analyzed by one-way ANOVA followed by Student's *t*-test. Quantitative data are presented as the mean ± SEM, whereas representative outcomes of the RT-PCR and immunohistochemical analyses are presented for qualitative assessment.
